# Retrospective Analysis of the Accuracy of High-Frequency Ultrasound for Pancreaticobiliary Maljunction in Pediatrics at a Single Center

**DOI:** 10.3389/fped.2022.775378

**Published:** 2022-04-14

**Authors:** Qiuchen Xu, Min Liu, Qiumei Wu, Wen Ling, Shan Guo

**Affiliations:** Department of Medical Ultrasonics, Fujian Maternity and Child Health Hospital, Affiliated Hospital of Fujian Medical University, Fuzhou, China

**Keywords:** ultrasonography, pancreaticobiliary maljunction (PBM), diagnostic imaging, congenital biliary dilatation, magnetic resonance cholangiopancreatography, endoscopic retrograde cholangiopancreatography, intraoperative cholangiography

## Abstract

**Objective:**

To determine the clinical value of high-frequency ultrasonography (US) in the evaluation and diagnosis of pancreaticobiliary maljunction (PBM) among children.

**Methods:**

The clinical subjects consisted of 31 pediatric patients who were diagnosed with PBM from January 2015 to May 2021 in Fujian Provincial Maternity and Children's Hospital. The primary outcomes included diagnosis accuracy, imaging characteristics of each type of PBM based on JSPBM, time length of operation, and cost of service. Secondary outcomes were the serum amylase and bilirubin levels.

**Results:**

The diagnostic accuracy of US was 90.3% and comparable to the other imaging methods—MRCP (82.6%), IOC (79.2%), and ERCP (100%), respectively. The time length of operation and direct cost were significantly lower than other imaging pathways. Stenotic type (A) is associated with a high internal diameter of CBD, and dilated channel type (C) presents increased internal diameter and length of CC as well as internal diameter of PD. There were higher levels of the serum bilirubin seen in type A and of serum amylase in type C compared with others.

**Conclusion:**

High-frequency US is a safe, cost-effective, and non-invasive imaging tool for the diagnosis and evaluation of PBM in pediatrics.

## Introduction

Pancreaticobiliary maljunction (PBM) is a congenital anomaly in which a junction of the pancreatic and bile ducts is anatomically outside the duodenal wall, forming a relatively long common channel above the sphincter of *Oddi*. Half a century ago, it was widely recognized as an abnormal union of biliopancreatic ducts or anomalous junction of the pancreaticobiliary ductal system (1). According to the presence or absence of the bile duct dilatation, PBM can be classified as PBM with congenital biliary dilatation and PBM without biliary dilatation (maximal diameter of the bile duct ≤ 10 mm).

In PBM, the regurgitation between the pancreatobiliary and biliopancreatic tract systems and premalignant changes in the gallbladder can occur due to loss of the regulation of the sphincter of *Oddi* (1–3). Patients with PBM have been found to have a high incidence of gallbladder and biliary tract cancers (4, 5). According to the report by a Japanese study (6), biliary cancer was seen in 21.6% of adult patients with congenital biliary dilatation and in 42.4% of patients with PBM without biliary dilatation. The cancer was located in the bile duct and gallbladder in between 32.1 and 62.3% of patients with dilatation and between 7.3 and 88.1% of patients with PBM without biliary dilatation. Moreover, these cancers presented 15–20 years earlier in patients with PBM than in patients without PBM. The incidence of biliary tract cancers in pediatric patients (younger than 15 years of age) with PBM is unknown, whereas a few case series/reports were published (7).

The main symptoms of PBM include abdominal pain, vomiting, jaundice, fever, and hyperamylasemia. Compared with patients with PBM without biliary tract dilatation, a larger proportion of patients with congenital biliary dilatation are symptomatic and related to the formation of the dilated duct. Many patients with neonatal/infant onset have been classified as showing cystic subtype in bile duct, in which main symptoms are jaundice and an abdominal mass. Those with the early onset version of the disease have a bile duct of a fusiform/cylindrical type and also present abdominal pain mostly due to increased intra-pancreatic/biliary pressure or due to the common channel being blocked by protein plugs. Generally, patients with non-dilated PBM often show symptoms in adulthood (8–10). Surgery is recommended for patients with PBM irrespective of the presence or absence of symptoms (7).

Abdominal ultrasonography (US) has conventionally been recommended as a primary imaging modality in the care of pediatric patients because of not only lower cost than other techniques, but also it being safety and being child-friendly. High-frequency ultrasound generally refers to the ultrasound probe frequency of more than 10 MHz. Because of its shorter wavelengths, it is absorbed easily for abdominal structures and, hence, is a reliable and non-invasive technique that is being used along with pediatric examination for assessment, diagnosis, and management. However, informative findings about the value of high-frequency US in the diagnosis of PBM have been limited. Although the development of imaging modalities including MRCP, ERCP, and IOC provided advantages for pediatric radiologists in early diagnosis of PBM, escalating medical costs is becoming more and more a concern in worldwide pediatric care clinics, suggesting a primary demand for pediatrics is reducing cost. We retrospectively analyzed our pediatric high-frequency US practice with diagnosis accuracy in clinical diagnosis of PBM. This study also explored the time length of operation and direct cost of different imaging tools in pediatric practice.

## Materials and Methods

### Patients

This study was approved by the Institutional Clinical Research Ethics Committee of Fujian Provincial Maternity and Children's Hospital. Written informed consent was obtained from the parents or guardians of all participants.

In this retrospective study, all pediatric patients with clinical features of biliary obstructive disease were included in the study. The US findings among 31 children (age <10 years) diagnosed with PBM between January 2015 and May 2021 at our Medical Center were analyzed. To confirm the diagnosis, we used the gold standard imaging tools—MRCP, IOC, and ERCP, which results were evaluated by radiologist blinded to the US findings. The morphologic characteristics of PBM were described according to the last classification proposed by JSPBM, which is based on the formation of the pancreaticobiliary junction (11). The four types are stenotic type (A) in which the distal common bile duct with stenosis joins the common channel, non-stenotic type (B) in which the distal common bile duct without stenosis joins the common channel, dilated channel type (C) in which the common channel is dilated, and complex type (D). The length of operation and the direct cost analysis in medical servings as high-frequency, MRCP, ERCP, and IOC were calculated on the basis of the updated standards issued by the national health and family planning administration.

The primary outcomes were diagnosis accuracy, imaging characteristics of each type of PBM, time length of operation, and cost of service. Secondary outcomes were the serum amylase and bilirubin levels associated with PBM types. The serum bilirubin level was determined on the basis of an orthodox diazo method, in which bilirubin reacts with diazotized sulfanilic acid to produce azobilirubin (purplein color). Intensity of color is directly proportional to the amount of bilirubin in the serum. To quantify the activity of amylase, the iodometric (amyloclastic) method was used to measure the rate at which substrate (starch) is reacted away. Iodine readily reacts with starch to produce a purple color, in which intensity is quantitatively associated with of the activity of amylase.

### High-Frequency US Technique

The high-frequency US facility was performed by the Voluson S8 scanner (GE Medical Systems, Waukesha, WI, USA) equipped with 9-MHz/11-MHz linear array transducers. All young patients underwent US imaging in the supine position within the sonography department at the hospital center. There was no additional force applied other than the weight of the probe to avoid any extra pain in the patients. During the operation, we applied a specific technique of graded compression to eliminate the disturbing impact of bowel gas. It allowed the use of a high-frequency probe for the assessment of the morphological abnormality by evaluating its reaction on compression. The compression should be applied slowly and gently to reduce abdominal pain. Four to five vertically oriented and overlapping lanes in the peritoneal cavity were needed to obtain better image quality using probes. The maximum inner diameters and lengths of the common bile duct, pancreatic duct, and common channel were reported by three experienced pediatric sonographers. The existence of a protein plug within the ductal system was observed. The electronic clinical records, sonography/radiology reports, and laboratory data of the children were analyzed for evidence of accuracy, sensitivity, and specificity in accordance with the other imaging diagnosis.

### Statistical Analysis

Data for continuous variables were analyzed for normality by Kolmogorov–Smirnov test and presented as mean ± SD. The *chi*-square or Fisher's exact tests were used to compare categorical variables and presented as mean and frequency with percentages. The normally distributed continuous variables were compared by unpaired *t*-test, whereas the others were tested by Mann–Whitney U test. Significant differences were defined as those with *p*-value< 0.05. All analyses were performed using SPSS statistical software (version 22).

## Results

### Clinical Characteristics

Of the 31 patients included in this study, there were 24 girls and 7 boys. The age distribution and clinical symptoms is shown in Table 1. Abdominal pain was the most common complaint (77.4%), followed by vomiting (32.3%), jaundice (22.6%), and fever (9.7%). One patient had chromosomal abnormality (*Down*'s syndrome) confirmed by karyotype test.

**Table 1:** Clinical characteristics of pediatrics with PBM.

		***N* = 31**
Gender	Girl	24 (77.4%)
	Boy	7 (22.6%)
Age (overall range)		2 days−9 years
**Clinical manifestation**		
Abdominal pain		24 (77.4%)
Vomiting		10 (32.3%)
Jaundice		7 (22.6%)
Fever		3 (9.7%)
*Down*'s syndrome		1 (3.2%)

### The Primary Outcomes

Among 31 cases of PBM, the vision of high-frequency US was read thoroughly by three sonographers. Further imaging with MRCP, IOC, or ERCP was considered to confirm the features suggestive of PBM. The overall diagnosis rate, time length of operation, and direct cost of service are shown in Table 2.

**Table 2:** Overall diagnosis rates, time length of operation, and cost of service in pediatrics with PBM.

	**US**			**MRCP**			**IOC**			**ERCP**		
	***N*** **=** **31**			***N*** **=** **23**			***N*** **=** **24**			***N*** **=** **9**		
	**DA**	**SE**	**SP**	**DA**	**SE^∧^**	**SP^∧^**	**DA**	**SE^#^**	**SP^#^**	**DA**	**SE^∧^**	**SP^∧^**
Diagnostic rate of PBM (%)	90.3	90.9	88.9	82.6	50–52.9	77.8–100	79.2	78.9	100.0	100.0	50–52.9	77.8–100
Time length of operation (min)	30 ± 4 ^*^			106 ± 19			176 ± 48			129 ± 31		
Cost ($/case)	34 ± 3.3 ^*^			270 ± 32			90 ± 10.2			388 ± 61		

*DA, diagnosis accuracy; SE, sensitivity; SP, specificity*;

* *p < 0.05 versus the other three tools. Based on the current sample studied, no expected SE/SP was available in groups of MRCP, IOC and ERCP*.

*The data were shown according to the literatures about ^∧^MRCP and ERCP applications in bile duct dilatation/occlusion and pancreatic duct occlusion/strictures (12), and ^#^IOC in bile duct injury (13)*.

Ultrasound accurately diagnosed PBM in 28 of 31 cases (90.3%) included in the study. Ultrasound was unable to diagnose a specific cause for three cases where false visualization in the common channel and common bile duct was read because of excessive intestinal gas. MRCP examination was performed in 23 cases, out of which 19 cases were accurately diagnosed PBM and four cases were false negative due to the very young age (2 days) with dilated CBD. IOC scan confirmed PBM in 19 of 24 children, whereas five were false negative due to failure to read common ducts (four cases) and being out of time as administration of the contrast agent drained into the duodenum (one case). ERCP was performed in nine cases, all of which diagnosed PBM accurately.

The snapshot of the time taken in imaging operation in diagnosis of PBM was summarized in Table 2. The high-frequency US procedure needed about half an hour in our clinical department. Over three times of US running operation time were needed in MRCP and ERCP examinations. IOC also needed a longer operation time compared with US measurement. Half of parents or guardians were concerned about the challenging issues from the medical imaging. Very few participants reported dissatisfactions or complaints just before/after the high-frequency US procedure.

Total per-patient direct costs in the medical center were $34 ± 3.3 for high-frequency US and $270 ± 32 for MRCP. IOC and ERCP services costed $90 ± 10.2 and $388 ± 61, respectively. We calculated that, as a result of administrating a high-frequency US examination, 11 (35.5%) MRCP and 13 (41.9%) IOC cases were avoided. The potential cost savings for use of high-frequency US in pediatric PBM instead of MRCP, IOC, and ERCP would be $236, 56, and 354 per examination.

### Ultrasonic Characteristics of PBM in Children

**Figure 1:**
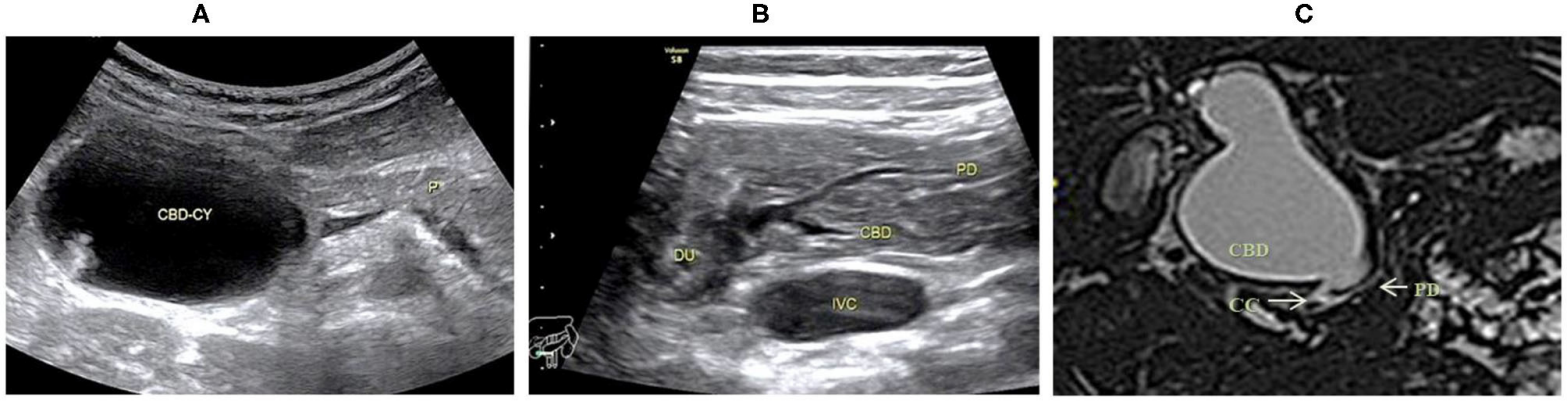
Images of a representative type A of PBM. **(A)** US transverse scan showed the dilated CBD containing the protein plugs; **(B)** US transverse scan showed the PD joining the terminal stenotic CBD within pancreas to form a common channel which drains into the duodenum; **(C)** MRCP indicated the dilated CBD with a narrow distal end joining the common channel.

**Figure 2:**
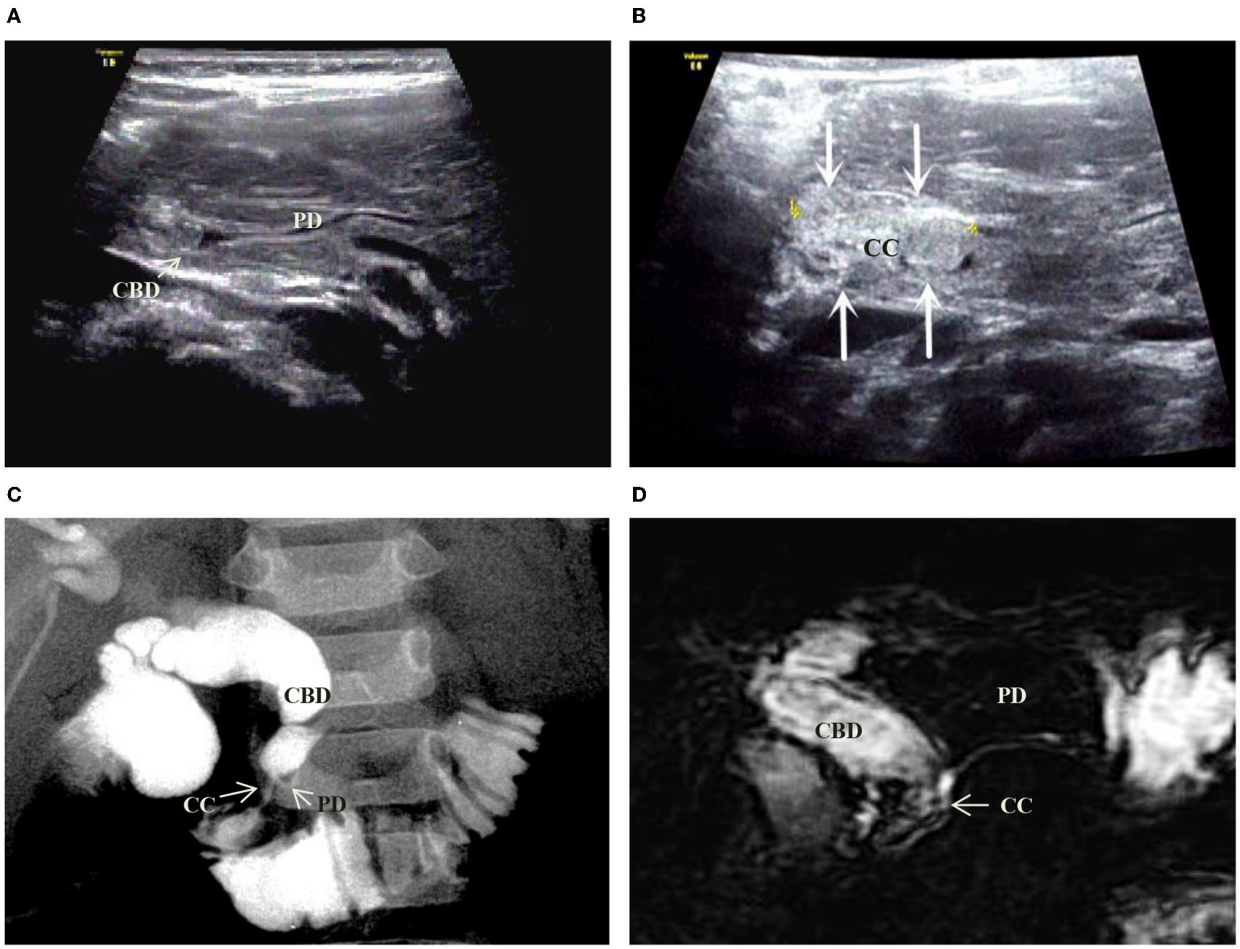
Images of a representative type B of PBM. **(A)** US transverse scan showed the PD (longitudinal section) joining the CBD which contains the protein plugs. **(B)** US oblique scan showed the common channel (between the arrows) is dilated and filled with protein plugs. **(C)** IOC indicated the PD and the dilated CBD joining at a right angle to form a common channel which inserts into the duodenum. **(D)** MRCP indicated the same features of PD and CBD as that in US and IOC. No significant narrowness in the distal CBD.

**Figure 3:**
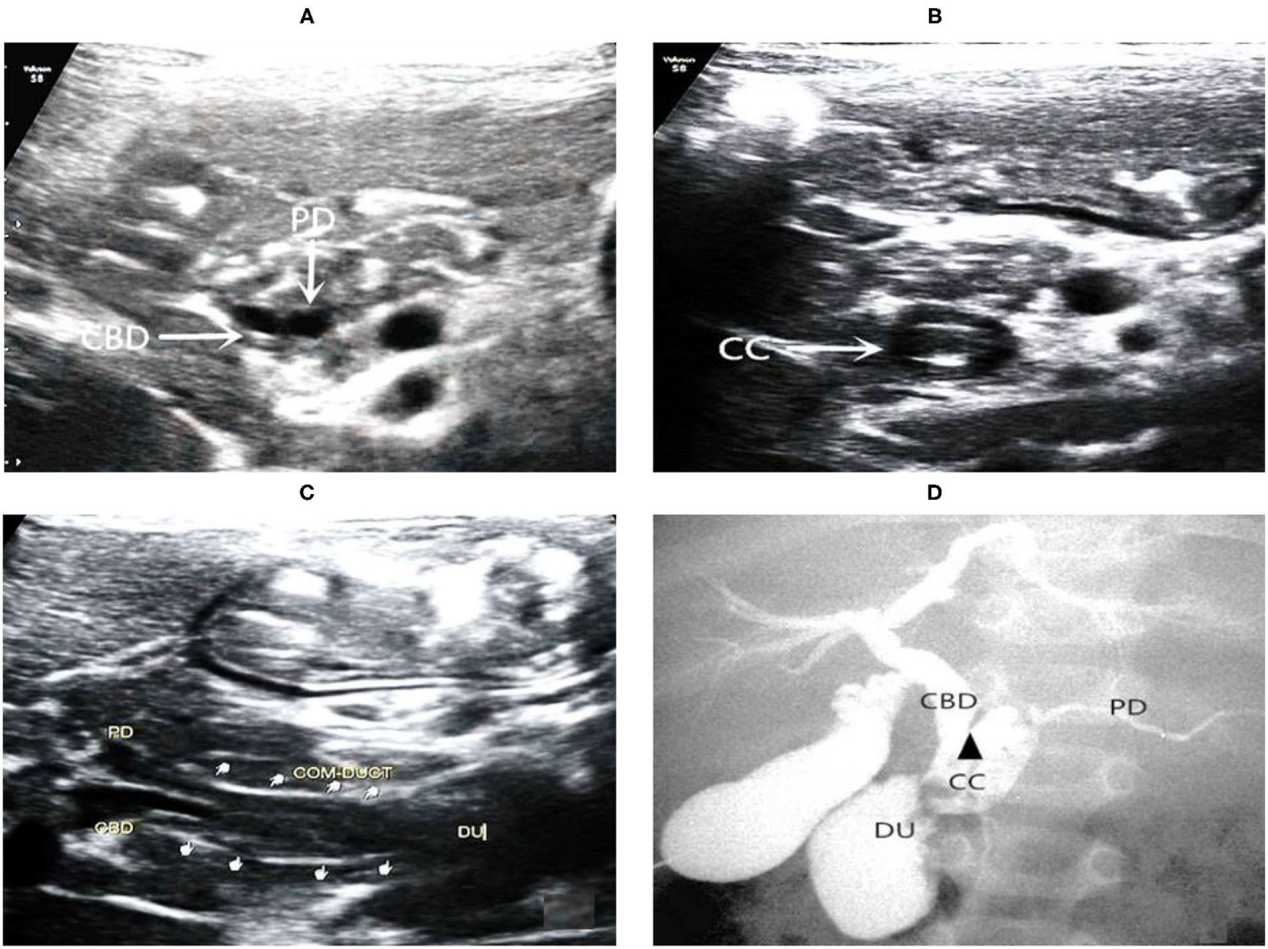
Images of a representative type C of PBM. **(A)** US transverse scan showed the axial section of both the terminal of the CBD and the PD; **(B)** US transverse scan showed the axial section of common channel and indicated protein plugs within; **(C)** US oblique scan showed the terminal CBD and the PD parallel each other and join at the end into the common channel which drains into the duodenum; **(D)** IOC confirmed dilations of the common channel and the CBD. There is an acute angle (arrow head) of interjunction of the PD and CBD.

**Figure 4:**
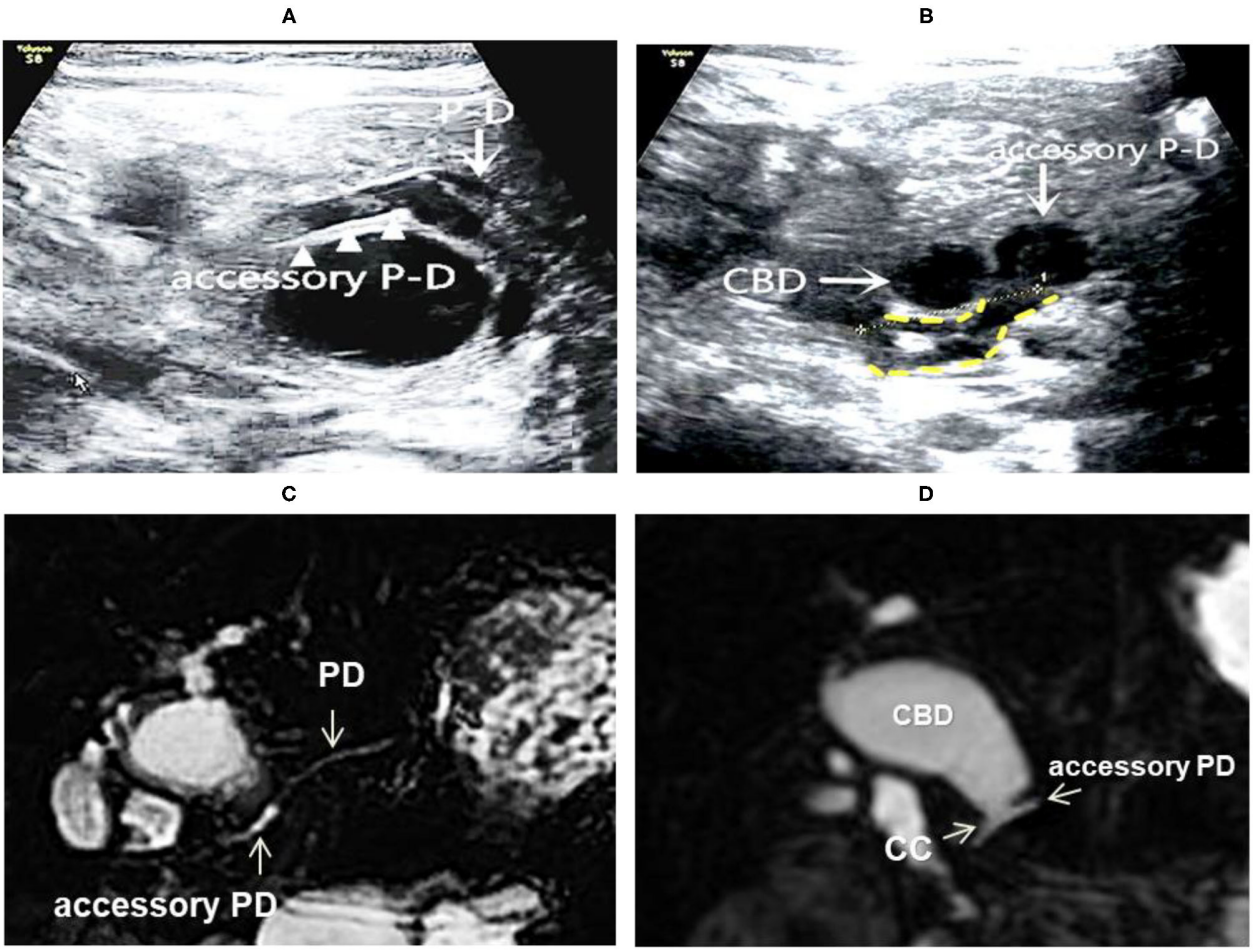
Images of a representative type C of PBM. **(A)** US transverses scan showing the main PD merging into the dilated accessory PD; **(B)** US transverse scan showing the terminal CBD and the accessory PD joining downward to form the common channel (shown by yellow dotted lines); **(C)** MRCP indicated the main PD imports to the accessary PD; **(D)** MRCP indicated the accessary PD joins with the distal CBD to form the common channel, suggesting a type D.

On the basis of the new JSPBM's classification, the individual morphologic features of PBM were described as four types (11) in presenting our ultrasound report: type A (stenotic)—the distal CBD with stenosis before joining with the CC, and an angle of slightly < 90° usually observed in the conjunction between PD and CBD at a site distant from the papilla of *Vater*; type B (non-stenotic)—the distal CBD emerging but without stenosis pattern before joining with the CC, in addition, an approximately right angle customarily seen in the conjunction between PD and CBD at a site distant from the papilla; type C (dilated channel)—the dilated CC significantly occurred; type D (complex)—a complicated configuration in PBJ contexture including the PD, the terminal portion of the CBD, and the dilated accessory PD, and one end opening as a small nipple in the duodenum and the other end converged with the CBD downward into the CC. Figures 1–4 show the representative imagines of A, B, C, and D types of PBM, respectively.

### Comparison of Morphological Classification

Among the 31 cases, 14 cases were stenotic type (A), 11 cased were non-stenotic (B), and five were dilated channel (C). The complex type (D) rarely occurred and only one case was found in our medical center. Table 3 shows the comparison among A, B, and C types in terms of gender, age, the maximum internal diameter of CBD, the internal diameter of CC, the length of CC, the internal diameter of the main PD, and the thickness of the gallbladder. Type A causes a significant increase in the internal diameter of CBD. Type C appears significant increases in internal diameter and length of CC and internal diameter of PD.

**Table 3:** Comparison of clinical data among the types (A, B, and C) of PBM.

**PBM type**	**A**	**B**	**C**
Girl/Boy	11/3	9/2	3/2
Age (range)	2 days−5 years	2–9 years	1.17–4.33 years
Internal diameter of CBD (mm)	26.43 ± 9.17^*^	12.66 ± 7.23	18 ± 8.17^#^
Internal diameter of CC (mm)	2.07 ± 0.87	4.23 ± 1.72	7.00 ± 2.11^*^
Length of CC (mm)	10.78 ± 2.25	11.82 ± 3.10	17.4 ± 3.73^*^
Internal diameter of PD (mm)	1.29 ± 0.45	1.74 ± 0.67	2.06 ± 0.79^*^
Thickness of gallbladder (mm)	2.27 ± 0.94	2.58 ± 0.95	2.48 ± 0.53
Serum bilirubin (μmol/L)	57.28 ± 23.19^*^	17.58 ± 7.87	15.26 ± 7.71
Serum amylase (U/L)	213.87 ± 48.24	245.17 ± 78.18	491.78 ± 151.30^*^
Bile amulase (U/L)	6,888.83 ± 2,250.88	7,247.03 ± 1,675.53	9,761.77 ± 2,200.40
Detective rate of protein plug (%)	85.7 (12/14)	100 (11/11)	100 (5/5)

*A, stenotic type; B, non-stenotic type; C, dilated channel type; CBD, common bile duct; CC, common duct*.

* *p < 0.05 vs. the other two types*.

# *P < 0.05 vs. type B*.

### The Secondary Outcomes

The serum bilirubin/amylase concentrations were shown in Table 3. Type A presented a higher level of serum bilirubin than other types, and the serum amylase level showed a significant higher in type C. All 31 children underwent extrahepatic bile duct resection and hepaticojejunostomy with Roux-en-Y anastomosis. No difference in biliary amylase levels was seen among the types (Table 3). The detective rates of protein plug were indicated individually among three types (Table 3), and the case of Type D was pointed out a positive image of protein plug in the US. The result showed that the overall detective rate of protein plug was 93.5%. The surgical treatment confirmed protein plugs in the distal segment of CBD and/or CC reported by preoperative US.

## Discussion

In the present study, we mainly aim to describe the current application of high-frequency US in the diagnosis of PBM and associated morphological findings in our pediatric center. Because of its higher resolution (16–158 μm) compared with ordinary ultrasound, high-frequency US was recognized as the ultrasound biomedical microscope (14, 15). In our investigation, its imaging qualities allow us clearly obtain the structure and thickness of ducts, channel, and gallbladder. Pediatric sonographers can diagnose the PBM on the basis of these results and can evaluate the follow-up treatment (data not shown). More importantly, our study suggests that high-frequency US is non-invasive, convenient, practical, and safe; therefore, it should gain more application in the field of pediatric PBM.

PBM is characterized by an abnormally long common channel and/or an abnormal union between the pancreatic and bile ducts. In 2020, the JSPBM proposed a new PBM classification, which is based on the formation of the PBJ especially the presence of dilatation in ductal structure (11). The four types classified by JSPBM provide us an actionable direction in early diagnosis of PBM with or without biliary dilatation by high-frequency US. Although the imaging methods used to demonstrate pancreatic and bile ducts remain direct cholangiography (ERCP, percutaneous trans-hepatic cholangiography, or IOC), MRCP, or three-dimensional drip infusion cholangiography computed tomography (1), clinical features of pediatric patients conceivably make quite difficult to support prevailing practice with the above golden standard tools. For example, ERCP requires general anesthesia and has potentially serious complications such as pancreatitis, hemorrhage, and bowel perforation. A recent study of 116 children demonstrated a morbidity of 3.4%, with a procedural failure rate of 2.5% (mainly due to the inability of major papilla catheterization) (16). Our practice suggests that ERCP operation is more difficult than sonography among infants and toddlers because the parents/guardians poorly accept ERCP operation for their children especially those <5 years. MRCP is a non-invasive, radiation-free, and safe procedure in examination of the pancreatic and liver parenchyma in addition to the ductal structures. However, the main disadvantage is limited spatial resolution especially in screening a PD <1 mm, compared with high-frequency US. In our study, the range of average internal diameter of the PD in children with PBM was 1.29 ± 0.45 to 2.06 ± 0.79 mm; additionally, MRCP hardly pinpointed a distinct exposure of pancreas in one case, overall resulting in a poor visualization and a lower accuracy (82.6%) than high-frequency US. Our findings are similar to other reports, which showed a 40–80% accuracy rate in diagnosis of PBM by MRCP (17–19). CT offers a higher resolution in abdominal contents than MRCP, but it has not been widely used in children due to exposure to ionizing radiation and usually iodinated contrast media, both of which have known safety concerns (20). MRI provides excellent evaluation of organ characteristics without exposure to ionizing radiation, but it has the difficulties in rendering a young patient sedation for the examination and the potential toxic influence of gadolinium-based contrast agents (21).

The abdominal wall of young children has thinner musculature and less abdominal adipose tissue than old children and adults; the solid organs are comparatively larger in young children than in adults, which indicates that more surface area is exposed, making the bile duct and pancreas accessible for high-frequency US to achieve. Although a low resolution has been associated with a depth of its scanning organs, high-frequency US did fulfill its detection among our young children (averaged 5 years old). Furthermore, high-frequency US is a non-invasive, radiation-free, high-resolution, and real-time dynamic technique that does not require sedation or have complications. One of the advantages in preoperative diagnosis of PBM by high-frequency US is that the scanning image not only can discern the abnormal confluence of the PD and the CBD but also further reveal the CC with slight rotation of the probe and continued examination to the extent possible. In addition, high-frequency US provides a satisfying measurement of the inner diameters of the ductal structures and visualizes protein plugs within, if present in the current study. For example, the overall detection rate of protein plugs is 95.2% (85.7–100%) in our study, which is higher than one reported for cholangiography and MRCP images (9.3–40%) (22, 23). The resulting difference displays that protein plugs are translucent in cholangiography and MRCP images but are detectable in high-frequency US examination, in which it presents acoustic images with homogeneous and moderately echoic characteristics. In the process of PBM diagnosis, because cholangiography or MRCP accurately showed the filling of contrast agent or liquid in the duct or channel, the protein plugs inside of it can falsely result in the illusion of lumen stenosis. In contrast, the high resolution of high-frequency US can truly clarify the inner diameter of the CBD/CC even with the presence of protein plugs.

Our study presents 90.9% in sensitivity by high-frequency US. The rate is lower than other report (24) with 100% in diagnosis of PBM. The difference could be possibly due to younger age in our study. Our results demonstrate a specificity of 88.9% on high-frequency US, which is similar with other studies with 88.4% in detecting the benign etiology of obstruction (25). There are a few potential reasons for false-negative ultrasound detection. One is that we could be able to overlook the inflamed PBJ. Other is the existence of air-filled dilated bowel loops that may hide ductal structure from view. Another pitfall is some demonstration of the part of ducts/channels within the scanning field because it is obscured or confused by multiple-sized protein plugs with inflamed tissues.

The fact that the US of abdominal organs is more susceptible to gastrointestinal gas was recognized in the investigation and would be most likely the one of reasons for false-negative detection by high-frequency US. Because of excessive intestinal gas in two cases, ultrasound visualization was limited to accessibility of the common channel and common bile duct, therefore a false reading occurring. To reduce the influence of the GI gas, some gentle compression should be recommended to pull way the bowel gas during the operation.

Our study indicated the financial and time advantages of using high-frequency US in pediatric PBM diagnosis, particularly in reducing the direct serving cost and cutting down time in imaging operation compared with the amount of other imaging tools performed. It is suggested that high-frequency US can be the recommended imaging modality of choice in pediatric PBM diagnosis.

Our study showed that the serum amylase concentration of patients with type C PBM was higher than other types of PBM. The possible explanation is that the dilated CC may have higher inner pressure against the pancreatic fluid upstream, or a smaller intersection angle of PD with CC is quite sharp to potentially obstruct the fluid from pancreas. However, further studies need to confirm the certain mechanisms on it.

### Limitations

The present study was limited by its retrospective nature and small sample size, e.g., type D. Some cases showed that PBJ region has poor visualizations on US, resulting in low-quality imagines.

## Conclusion

High-frequency US is a safe and cost-effective diagnostic tool for suspected cases of PBM in children. Apart from the diagnosis, it can provide valuable information about the type of PBM and the ductal structure as well as protein plugs.

## Data Availability Statement

The original contributions presented in the study are included in the article/supplementary material, further inquiries can be directed to the corresponding author/s.

## Ethics Statement

The studies involving human participants were reviewed and approved by Institutional Clinical Research Ethics Committee of Fujian Provincial Maternity and Children's Hospital: https://www.natureindex.com/institution-outputs/china/fujian-provincial-maternity-and-children-s-hospital/5df1dfe3051f67680f677c40. Written informed consent to participate in this study was provided by the participants' legal guardian/next of kin.

## Author Contributions

ML, QW, and WL: data collection. QX: draft manuscript preparation. All authors reviewed the results and approved the final version of the manuscript.

## Conflict of Interest

The authors declare that the research was conducted in the absence of any commercial or financial relationships that could be construed as a potential conflict of interest.

## Publisher's Note

All claims expressed in this article are solely those of the authors and do not necessarily represent those of their affiliated organizations, or those of the publisher, the editors and the reviewers. Any product that may be evaluated in this article, or claim that may be made by its manufacturer, is not guaranteed or endorsed by the publisher.

## Abbreviations

PBM: Pancreaticobiliary maljunction
US: Ultrasonography
JSPBM: Japanese Study Group on Pancreaticobiliary Maljunction
MRCP: Magnetic resonance cholangiopancreatography
ERCP: Endoscopic retrograde cholangiopancreatography
IOC: Intraoperative cholangiography
CBD: Common bile duct
PD: Pancreatic duct
CC: Common duct
PBJ: Pancreaticobiliary junction
DU: Duodenum.

## References

[1] Kamisawa T Ando H Hamada Y Fujii H Koshinaga T Urushihara N. Diagnostic criteria for pancreaticobiliary maljunction 2013. J Hepatobiliary Pancreat Sci. (2014) 21:159–61. 10.1002/jhbp.57 24307541

[2] Ono S Fumino S Iwai N. Diagnosis and treatment of pancreaticobiliary maljunction in children. Surg Today. (2011) 41:601–5. 10.1007/s00595-010-4492-9 21533929

[3] Guo WL Huang SG Wang J Sheng M Fang L. Imaging findings in 75 pediatric patients with pancreaticobiliary maljunction: a retrospective case study. Pediatr Surg Int. (2012) 28:983–8. 10.1007/s00383-012-3159-6 22892909 PMC3445796

[4] Kamisawa T Kuruma S Chiba K Tabata T Koizumi S Kikuyama M. Biliary carcinogenesis in pancreaticobiliary maljunction. J Gastroenterol. (2017) 52:158–63. 10.1007/s00535-016-1268-z 27704265

[5] Ragot E Mabrut JY Ouaïssi M Sauvanet A Dokmak S Nuzzo G. Pancreaticobiliary maljunctions in European patients with bile duct cysts: results of the multicenter study of the French Surgical Association (AFC). World J Surg. (2017) 41:538–45. 10.1007/s00268-016-3684-x 27620132

[6] Kamisawa T Ando H Shimada M Hamada Y Itoi T Takayashiki T. Recent advances and problems in the management of pancreaticobiliary maljunction: feedback from the guidelines committee. J Hepatobiliary Pancreat Sci. (2014) 21:87–92. 10.1002/jhbp.8 23798483

[7] Le Roy B Gagnière J Filaire L Fontarensky M Hordonneau C Buc E. Pancreaticobiliary maljunction and choledochal cysts: from embryogenesis to therapeutics aspects. Surg Radiol Anat. (2016) 38:1053–60. 10.1007/s00276-016-1669-y 27003810

[8] Tashiro S Imaizumi T Ohkawa H Okada A Katoh T Kawaharada Y. Pancreaticobiliary maljunction: retrospective and nationwide survey in Japan. J Hepato Biliary Pancreatic Surg. (2003) 10:345–51. 10.1007/s00534-002-0741-7 14598134

[9] Iwai N Fumino S Tsuda T Ono S Kimura O Deguchi E. Surgical treatment for anomalous arrangement of the pancreaticobiliary duct with nondilatation of the common bile duct. J Pediatr Surg. (2004) 39:1794–6. 10.1016/j.jpedsurg.2004.08.010 15616932

[10] Ohuchida J Chijiiwa K Hiyoshi M Kobayashi K Konomi H Tanaka M. Long-term results of treatment for pancreaticobiliary maljunction without bile duct dilatation. Arch Surg. (2006) 141:1066–70. 10.1001/archsurg.141.11.1066 17116798

[11] Ono A Arizono S Isoda H Togashi K. Imaging of pancreaticobiliary maljunction. Radiographics. (2020) 40:378–92. 10.1148/rg.2020190108 31951513

[12] Hurter D De Vries C Potgieter P Barry R Botha F Joubert G. Accuracy of MRCP compared to ERCP in the diagnosis of bile duct disorders. SA J Radiology. (2008) 12:14–22.

[13] Alvarez FA De Santibañes M Palavecino M Sánchez Clariá R Mazza O Arbues G. Impact of routine intraoperative cholangiography during laparoscopic cholecystectomy on bile duct injury. J Bri Surg. (2014) 101:677–84. 24664658 10.1002/bjs.9486

[14] Jasaitiene D Valiukeviciene S Linkeviciute G Raisutis R Jasiuniene E Kazys R. Principles of high-frequency ultrasonography for investigation of skin pathology. J Eur Acad Dermatol Venereol. (2011) 25:375–82. 10.1111/j.1468-3083.2010.03837.x 20849441

[15] Petrella LI de Azevedo Valle H Issa PR Martins CJ Machado JC Pereira WC. Statistical analysis of high frequency ultrasonic backscattered signals from basal cell carcinomas. Ultrasound Med Biol. (2012) 38:1811–9. 10.1016/j.ultrasmedbio.2012.06.001 22920547

[16] Morine Y Shimada M Takamatsu H Araida T Endo I Kubota M. Clinical features of pancreaticobiliary maljunction: update analysis of 2nd Japan-nationwide survey. J Hepato Biliary Pancreatic Sci. (2013) 20:472–80. 10.1007/s00534-013-0606-2 23579999

[17] Varadarajulu S Wilcox CM Hawes RH Cotton PB. Technical outcomes and complications of ERCP in children. Gastrointest Endosc. (2004) 60:367–71. 10.1016/S0016-5107(04)01721-3 15332025

[18] Kim MJ Han SJ Yoon CS Kim JH Oh JT Chung KS. Using MR cholangiopancreatography to reveal anomalous pancreaticobiliary ductal union in infants and children with choledochal cysts. Am J Roentgenol. (2002) 179:209–14. 10.2214/ajr.179.1.1790209 12076938

[19] Matos C Nicaise N Deviere J Cassart M Metens T Struyven J. Choledochal cysts: comparison of findings at MR cholangiopancreatography and endoscopic retrograde cholangiopancreatography in eight patients. Radiology. (1998) 209:443–8. 10.1148/radiology.209.2.9807571 9807571

[20] Doria AS Moineddin R Kellenberger CJ Epelman M Beyene J Schuh S. US or CT for diagnosis of appendicitis in children and adults? A meta-analysis. Radiology. (2006) 241:83–94. 10.1148/radiol.2411050913 16928974

[21] Malviya S Voepel-Lewis T Eldevik OP Rockwell DT Wong JH Tait AR. Sedation and general anaesthesia in children undergoing MRI and CT: adverse events and outcomes. Br J Anaesth. (2000) 84:743–8. 10.1093/oxfordjournals.bja.a013586 10895749

[22] Chapuy S Gorincour G Roquelaure B Aschero A Paris M Lambot K. Sonographic diagnosis of a common pancreaticobiliary channel in children. Pediatr Radiol. (2006) 36:1300–5. 10.1007/s00247-006-0322-z 17028852

[23] Kaneko K Ando H Ito T Watanabe Y Seo T Harada T. Protein plugs cause symptoms in patients with choledochal cysts. Am J Gastroenterol. (1997) 92:1018–21. 9177522

[24] Huang SG Guo WL Wang J Sheng M Lan XH Fang L. Factors interfering with delineation on MRCP of pancreaticobiliary maljunction in paediatric patients. PLoS ONE. (2016) 11:e0154178. 10.1371/journal.pone.0154178 27104956 PMC4841599

[25] Kushnir VM Wani SB Fowler K Menias C Varma R Narra V. Sensitivity of endoscopic ultrasound, multidetector computer tomography and magnetic resonance cholangiopancreatography in the diagnosis of pancreas divisum: a tertiary center experience. Pancreas. (2013) 42:436. 10.1097/MPA.0b013e31826c711a 23211370 PMC3928633

